# The Inflammatory Role of Pro-Resolving Mediators in Endometriosis: An Integrative Review

**DOI:** 10.3390/ijms22094370

**Published:** 2021-04-22

**Authors:** Cássia de Fáveri, Paula M. Poeta Fermino, Anna P. Piovezan, Lia K. Volpato

**Affiliations:** 1 Medical Residency Program in Ginecology and Obstetric, Hospital Regional Dr. Homero Miranda Gomes, São José 88103-901, Brazil; cassia_faveri@hotmail.com; 2 Department Curso de Medicina, Campus Pedra Branca, Undergraduate Medical School, Universidade Sul de Santa Catarina—UNISUL, Palhoça 88137-272, Brazil; paulampf@icloud.com; 3 Postgraduate Studies in Health Science Program, Universidade do Sul de Santa Catarina—UNISUL, Palhoça 88137-272, Brazil; Anna.Piovezan@unisul.br; 4 Ginecology and Obstetric Department, Hospital Regional Dr. Homero Miranda Gomes, São José 88103-901, Brazil

**Keywords:** endometriosis, inflammation mediators, annexin A1, Lipoxin A4, receptors, Lipoxin, Resolvin, review

## Abstract

The pathogenesis of endometriosis is still controversial, although it is known that the inflammatory immune response plays a critical role in this process. The resolution of inflammation is an active process where the activation of endogenous factors allows the host tissue to maintain homeostasis. The mechanisms by which pro-resolving mediators (PRM) act in endometriosis are still little explored. Thus, this integrative review aims to synthesize the available content regarding the role of PRM in endometriosis. Experimental and in vitro studies with Lipoxin A4 demonstrate a potential inhibitory effect on endometrial lesions’ progression, attenuating pro-inflammatory and angiogenic signals, inhibiting proliferative and invasive action suppressing intracellular signaling induced by cytokines and estradiol, mainly through the FPR2/ALX. Investigations with Resolvin D1 demonstrated the inhibition of endometrial lesions and decreased pro-inflammatory factors. Annexin A1 is expressed in the endometrium and is specifically present in women with endometriosis, although the available studies are still inconsistent. Thus, we believe there is a gap in knowledge regarding the PRM pathways in patients with endometriosis. It is important to note that these substances’ therapeutic potential is evident since the immune and abnormal inflammatory responses play an essential role in endometriosis development and progression.

## 1. Introduction

Endometriosis is a chronic, inflammatory and estrogen-dependent disease characterized by endometrial tissue outside the uterine cavity. It affects approximately 10% of women of reproductive age and is associated with chronic pelvic pain and infertility [1]. The pathophysiology of endometriosis is controversial. Sampson’s (1927) retrograde menstruation theory is still the most accepted, describing that the reflux of endometrial fragments would allow the implantation of these cells outside the uterus, especially in the pelvic cavity [2]. However, genetic, neuronal, hormonal and immunological variations may facilitate the adhesion and development of endometrial implants [3].

It is known that 17-β-estradiol (E2) plays a strong influence on the development and progression of endometriosis, acting via estrogen receptors (ER) that are abundant in reproductive tissues, in addition to activating several intracellular signaling cascades in the inflammatory process [4].

Several studies demonstrate that the immune response plays an essential role in the genesis of endometriosis. Many active immune cells, especially peritoneal macrophages, are involved in the development, maintenance and progression of endometrial lesions [1]. High concentrations of cytokines, growth factors and angiogenic factors are observed in the peritoneal fluid of subjects with endometriosis [5]. Interleukins, tumor necrosis factors (TNF) and other chemotactic cytokines act as recruiting macrophages and T lymphocytes to the peritoneum, modulating the inflammatory response associated with endometriosis [6]. Among the factors that support the invasive and proliferative activity of endometrial implants, the epithelial-mesenchymal transition (EMT) is characterized as a biological process in which cells lose cell polarity cell-cell adhesion, and gain migratory and invasive properties to become mesenchymal stem cells [7]. Additionally, matrix metalloproteinases (MMPs) act out as cell-matrix remodeling enzymes that induce the release of growth factors and pro-inflammatory cytokines, favoring the progression of inflammation angiogenesis, tissue remodeling and, therefore, contributing to the implantation and increase of endometriotic lesions [8].

Furthermore, cellular signaling proteins such as p38 MAPK (p38 mitogen-activated protein kinases) and ERK (extracellular signal-regulated kinase) are critical in the inflammatory response. They are characterized as intracellular signal transduction molecules activated by phosphorylation via membrane receptors, increasing levels of cytokines such as interleukins, and TNF, in addition to increasing MMP activity. In addition, several studies show its influence on the pathogenesis of endometriosis [9]. The multidrug resistance-associated protein 4 (MRP4) can transport several endogenous molecules, playing a critical role in cellular communication and signaling. Among these molecules, there is a strong affinity with Prostaglandin E2 (PGE2), consequently potentiating the inflammatory process [10].

Several inflammatory response events can limit their magnitude and duration [5]. The resolution of inflammation is an active process where the activation of endogenous factors allows the host tissue to maintain homeostasis [11]. This process occurs differently concerning the known anti-inflammatory pathways since the pro-resolving molecules act in a multifactorial way at the inflammatory site. In addition to producing powerful signals to reduce neutrophils and eosinophils’ infiltration, they also promote the uptake and elimination of apoptotic cells and microorganisms by macrophages at the inflamed tissue. At the beginning of the inflammatory process, classic lipid mediators such as prostaglandins and leukotrienes are released, activating and amplifying the inflammatory process. After the acute phase, some of these molecules start to produce substances that synthesize endogenous mediators with anti-inflammatory and pro-resolving activity, such as lipoxins, resolvins, protectins and maresines [12,13,14].

The pro-resolving mediators (PRM) are lipids or proteins. Lipid mediators are generated through lipoxygenases (LOX) and cyclooxygenases (COX) by the metabolism of arachidonic acid (AA), such as lipoxins, or originated from omega-3 polyunsaturated fatty acids (omega-3 PUFAS), represented by the acid eicosapentaenoic (EPA)-derived resolvin E (RvE) and docosahexaenoic acid (DHEA)-derived resolvin D (RvD), protectins and maresines. These mediators promote the sequestration of pro-inflammatory cytokines, in addition to removing polymorphonuclear cells (PMN) from the epithelial surface, phagocyting apoptotic PMNs, removing inflammatory residues through the lymphatic vessels and reducing inflammatory pain [15,16,17].

In endometriosis, the signaling pathways for Lipoxin A4 (LXA4) are the most studied. Its cascades are mediated by several membrane receptors [18] with higher affinity for the formyl peptide 2/”aspirin-triggered lipoxin” receptor (FPR2/ALX); therefore, most studies show how LXA4 acts through this receptor [19], allowing it to act in a double, anti-inflammatory and pro-resolutive manner [12]. Some works have already shown that LXA4 presents a high structural similarity with estriol, a weak agonist of the ER-α in the endometrium’s epithelial cells. For this reason, LXA4 can also occupy these receptors and decrease E2-mediated signaling, triggering anti-inflammatory and pro-resolving effects, in addition to modulating the expression of ERs [4,18,20].

The PRM proteins are Annexin A1 (ANXA1), galectins and melanocortins. These mediators have a crucial modulating function in neutrophil trafficking. They can reduce infiltration, activate apoptosis at the inflammatory site, stimulate phagocytosis and the elimination of apoptotic neutrophils, in addition to inducing the phenotypic change from inflammatory M1 macrophages to M2 anti-inflammatory macrophages, which causes a reparative response [17,21,22]. ANXA1 is a calcium-dependent phospholipid-binding protein and has been observed as an anti-inflammatory mediator, regulating physiological and pathological cellular processes. Additionally, ANXA1 is expressed in several tissues, including the endometrium, where it acts via the FPR2/ALX and other mediators [23].

The FPR2/ALX regulates the action of PRM, such as ANXA1 and LXA4, belonging to the superfamily of formyl peptide receptors. These receptors are critical in endometriosis since the expression of FPR2/ALX proved to be more significant in the cells of endometriotic lesions compared to the normal endometrium. In addition, these receptors are regulated by estrogen and other cytokines and mediate specific cellular pathways to suppress inflammation [18,23].

Failures in these pro-resolving pathways can predispose the host to chronic inflammatory diseases [12]. In this process, cellular mechanisms and their biochemical pathways open new strategies for potential therapeutic interventions [24]. Evidence that PRM are promising targets for the development of pharmacological treatments for chronic inflammatory diseases is pointed out by Serhan (2017); in this, the author demonstrates examples of conditions for which drugs like PRM have been successfully studied in clinical trials in humans (phase I and phase II), including periodontal diseases, inflammation associated with dry eye and childhood eczema [25].

The mediators that act as PRM in endometriosis are still little explored. Thus, this review aims to synthesize the information available in the literature regarding the inflammatory role of PRM in endometriosis.

## 2. Methods

### 2.1. Search Strategy

For this integrative review, a survey of all articles published and indexed in the main known databases was conducted: MEDLINE (PubMed), Bireme (LILACS, ADOLEC, IBECS and BDENF), EMBASE (Elsevier) and DOAJ. The search was carried out between June and August 2020, without restrictions regarding the date limit of publication or language. The terms “endometriosis”, “pro-resolution mediators”, “lipoxin”, “maresin”, “resolvin”, “protectin”, “FPR”, “Annexin A1”, “galectin” and “melanocortin” were used, and additionally the Boolean operators “AND” and “OR” applied for “endometriosis” and the other terms, respectively.

### 2.2. Articles Eligibility Criteria

The eligibility criteria were applied to the selected articles with support of the reference manager software Mendeley© Version 1.19.4 (Mendeley Ltd., London, UK), by which duplicates were identified and excluded. Two independent researchers screened the articles by reading the title and abstract. Full-length original articles whose theme covered the role of PRM in endometriosis by experimental models, cell culture or in humans were included. Articles written in an alphabet other than the Roman alphabet and review articles were excluded.

### 2.3. Data Processing

After the screening, two independent researchers performed the data extraction by filling out a clinical form in a previously established spreadsheet. Finally, the findings were analyzed and the written data descriptively synthesized.

## 3. Results and Discussions

**Figure 1:**
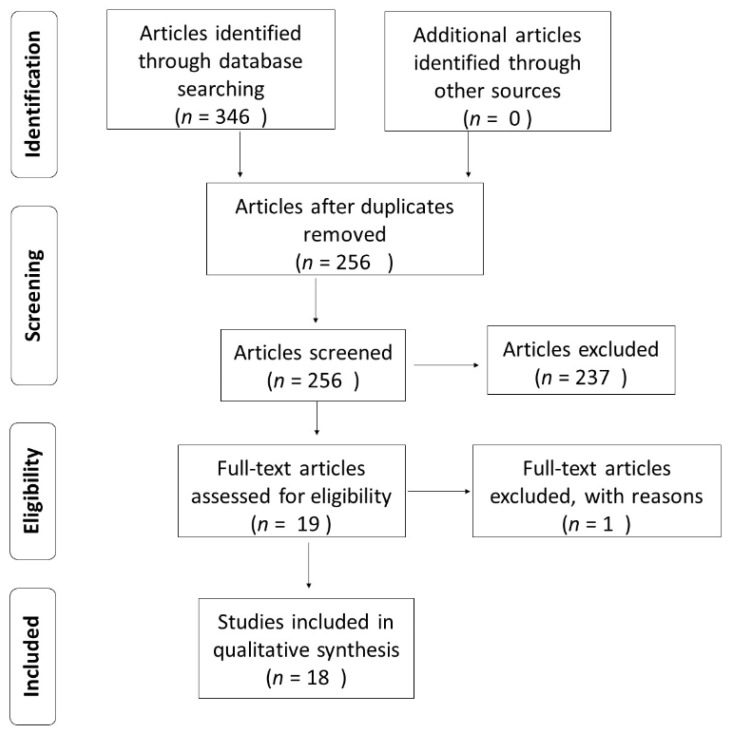
Flowchart of article selection. São José-SC—Brazil, 2021.

In the initial search, 336 articles were identified, 90 of which were duplicates, resulting in 246 articles. The studies were screened by reading the title and abstract, with 19 articles selected and one excluded since the full-length text was only available in the Mandarin language (Figure 1). The distribution of the captured articles and the related terms are shown in Table 1. No research was found addressing the terms “protectin”, “galectin” or “melanocortin” associated with “endometriosis”. The included studies and their main findings are shown in Table 2.

### 3.1. Methods Used in the Studies Reviewed

#### 3.1.1. Experimental Studies: Methods to Induce and Evaluate Endometriosis

In experimental studies, endometriosis is induced in mice with transplantation of endometrial fragments in the peritoneal cavity by autologous or heterologous transplantation of the uterine horns. The endometrium-rich fragments are obtained by removing the uterine corn from the donor or through minor surgery to collect endometrial tissue and transplant it in the same animal. The fragments are injected or implanted in the recipient animals’ peritoneal cavity. The animals have randomly grouped accordingly: those submitted to endometriosis that received treatment, those submitted to endometriosis without treatment and a control group not submitted to experimental endometriosis. Following the treatment with the respective mediator, the mice are sacrificed or re-operated to assess the lesions and collect the tissue to measure the intervention effects [7,19,26,28,29,32,35,40,41].

Some studies have also carried out hormonal stimulation of endometrial lesions with estrogen administration [29] or estrogen and 17-OH-progesterone [26]. In Tomio et al. investigation, donor and recipient were oophorectomized and stimulated with estrogen [31]. In addition, researchers have used vaginal cytology in mice to define its estrous cycle stage [27,28,29,41]. Further, Dmitrieva et al. injected Evans Blue dye to assess leakage in endometrial lesions and implant a telemetric probe to analyze vaginal nociception in mice [30].

#### 3.1.2. In Vitro Studies

In vitro studies were carried out using primary endometriotic stromal cells (ESCs) collected and isolated from endometriotic lesions or cells from patients’ eutopic endometrium endometriosis diagnosed by surgery and histological process. The disease stage was established according to the American Society for Reproductive Medicine (ASRM). Control group patients were confirmed with the absence of endometriosis; tissue cells from the peritoneal cavity were collected when operated due to other reasons. In these studies, patients were at least 3–6 months without hormonal or anti-inflammatory treatment. ESCs were treated in cell culture with the mediator under investigation, and the evaluation was carried out through several types of laboratory analysis [7,19,30,31,33,34,35]. Rasheed et al. analyzed in vitro halogenic stem cells by adding serum from patients diagnosed with endometriosis to evaluate cell differentiation and the expression of ANXA1 [37].

#### 3.1.3. Cross-Sectional Studies in Humans

Cross-sectional studies evaluated biopsies of endometrial lesions and peritoneal fluid in patients with endometriosis who underwent surgery. The disease stage was classified according to criteria established by the ASRM. Additionally, patients in the control group were confirmed with the absence of endometriosis and tissue cells from the peritoneal cavity collected when operated on due to other reasons. Then, the study was conducted based on an analysis of different types of laboratory exams [26,31,36,37,38,39].

### 3.2. PRM and Endometriosis

#### 3.2.1. Lipoxin A4

##### LXA4 Inhibits the Progression of Endometrial Lesions in Experimental Studies

According to the studies evaluated here, treatment with LXA4 or the analog 15-epi-LXA4 performed according to the experimental model previously mentioned does not alter the mice’s estrous cycle or ovarian function. Therefore, it does not prevent the development of endometriosis [27,28,29]. However, it inhibits established lesions’ progression by significantly reducing endometriotic lesions’ size and weight that histologically present a rudimentary architecture, with less glandular and stromal development [7,19,27,28,29,32,35].

##### LXA4 Attenuates Pro-Inflammatory and Angiogenic Effects Associated with Endometriosis

Evidence shows that LXA4 attenuates pro-inflammatory and angiogenic effects associated with endometriosis. In an experimental study, LXA4 reduced the expression of COX-2 [32,35] and PGE2 levels in endometriotic lesions and peritoneal fluid cells [32]. LXA4 reduced pro-inflammatory cytokines in lesions, ESCs and peritoneal fluid cells during in vivo and in vitro experiments. Among the studied cytokines, treatment with LXA4 reduced the expression of interleukine 1β (IL-1β), interleukin 6 (IL-6), interleukin 10 (IL-10), interleukin 16 (IL-16), vascular endothelial growth factor (VEGF), TNF, transforming growth factor (TGF)-β1 and TGF-β2 [19,27,29,31,32,33].

##### LXA4 Suppresses MMP Activity, Proliferative Action and Cell Cycle Progression

In experimental models of endometriosis, evidence has shown that LXA4 suppresses the activity of MMPs (MMP-9 and MMP-2) in endometrial lesions via the FPR2/ALX in mice [7,28,29,32]. Treatment with LXA4 in vitro also inhibited the cell cycle’s progression in ESCs, consequently attenuating the invasive and proliferative activity associated with endometriotic lesions [19].

##### LXA4 Modulates the Expression of Estrogen Receptors

A cross-sectional study of ESCs has shown that treatment with LXA4 and E2 triggered a higher ER-β expression and decreased pro-inflammatory signals. The authors suggest that LXA4 may selectively modulate ER-β in ESCs. In addition, the human endometrium analysis showed a strong positive correlation between the expression of LXA4 and ER-α. In contrast, a negative correlation with ER-β was observed in an in vitro analysis. Further, there was no association between LXA4 and the progesterone receptor (RP). Despite the divergence, it is evident that LXA4 regulates ERs by attenuating E2-induced inflammatory signaling pathways in addition to acting as an E2 receptor agonist [31].

##### LXA4 Suppresses Cell Signaling, p38 MAPK and ERK Phosphorylation Induced by Estrogen in ESCs

Another in vitro effect elicited by LXA4 in ESCs is reducing E2-induced p38 MAPK and ERK phosphorylation. Important, estradiol is known to stimulate the activity of these enzymes. Additionally, E2-induced p38 MAPK phosphorylation is significantly reduced in cells treated with E2 and LXA4, suggesting that LXA4 may inhibit endometriosis development by this route, probably through ERs [31]. Further, the inhibition of E2-induced p38 MAPK and ERK phosphorylation is mediated by the FPR2/ALX in vitro [7]. In a proteomic analysis, the combined treatment with E2 and LXA4 resulted in reduced regulated proteins, with LXA4 mediating a suppressive effect on E2-mediated inflammatory cell signaling [33].

##### LXA4 Suppresses E2-Induced Epithelial-Mesenchymal Transition

LXA4 suppressed E2-induced EMT of ESCs in vitro, reversing in a dose-dependent manner the reduced expression of epithelial markers (E-cadherin) and the increased expression of mesenchymal markers (Vimentin, N-cadherin and Zinc Finger E-box-binding homeobox 1 (ZEB1)) induced by E2, thus preventing the progress, migration and invasion promoted by endometriosis [7].

##### LXA4 Reduces p38 MAPK Phosphorylation, Cytokine Release and COX-2 Expression Induced by Interleukin-1β in ESCs via FPR2/ALX

IL-1β is an important pro-inflammatory cytokine that stimulates other cytokines and angiogenic factors. Important to note, LXA4 inhibited IL-1β-induced cytokine release, additionally also inhibited IL-1β-induced p38 MAPK phosphorylation via FPR2/ALX in an in vitro assay [19,35]. Further, LXA4 inhibited COX-2 expression induced by IL-1β in ESCs through the FPR2/ALX modulation. According to Wu et al. in proteomic analysis, LXA4 can suppress proteins that facilitate IL-1β-induced migration and invasion in ESC [34].

##### LXA4 Attenuates MRP4 Expression in ESCs

Gori et al. first described the expression of MRP4 in the human endometrium, showing increased peritoneal endometriotic lesions associated with high levels of PGE2 in the peritoneal fluid. The same study showed that LXA4 significantly attenuated the expression of MRP4 in vitro, thus disabling intracellular signals associated with inflammation in endometriosis [30].

#### 3.2.2. Resolvins

##### Resolvin D1 (RvD1) Decreases Inflammatory Signs Associated with Endometriosis

The treatment with RvD1 and the analog 17(R)-RvD1 decreased the inflammatory signs of vascular permeability and neurogenic activity as reflected by the significant reduction of Evans Blue dye leakage in ectopic lesions in mice submitted to the experimental model previously mentioned. Moreover, RvD1 effectively reduced vaginal hyperalgesia in mice, proving its potential to relieve abnormal endometriosis-related pelvic pain [41].

##### Resolvin E3 (RvE3) Inhibits the Progression of Endometriosis

In an experimental study, 15/12-LOX-KO mice (animals with 15/12 lipoxygenase enzyme deficiency) and wild mice submitted to endometriosis received oral EPA to investigate the effects of 12/15-LOX-related mediators in endometriotic lesions and compared with wild mice subjected to endometriosis which did not receive oral EPA. The administration of EPA significantly decreased the number of endometriotic lesions in wild mice, although the suppressive effect of EPA on the development of endometriotic lesions was not observed in 12/15-LOX-KO mice. In addition, the number of endometriotic lesions was similar in 12/15-LOX-KO mice treated or not with EPA. Interestingly, the EPA-derived bioactive mediator 18S/R-RvE3, which was biosynthesized from 18-HEPE (hydroxyicosapentaenoic acid) by 15/12-LOX was increased in the peritoneal fluid of wild mice following the administration of EPA. However, this increase was not observed in 12/15-LOX-KO mice, suggesting that RvE3 may be involved in modulating endometriotic lesions [40].

#### 3.2.3. Annexin A1

##### Expression of ANXA1 Protein in Endometriosis

Cross-sectional studies have assessed the role of ANXA1 in the pathogenesis of endometriosis in humans. It was shown that its expression is more significant in the endometrium of women with endometriosis [36] and higher in endometriotic lesions (abdominal wall endometrioma). Furthermore, the high levels correlate with morphological changes in this tissue, suggesting that ANXA1 may be involved in cell differentiation and proliferation [38]. However, these data are inconsistent since another study showed a reduced expression of ANXA1 in patients with endometriosis, suggesting that this decrease can facilitate the inflammatory process [39]. In this sense, the lack of studies to better assess the role of ANXA1 and its pathways in endometriosis is evidenced.

##### ANXA1 as Endometriosis Inducing Factors

The involvement of predisposing factors in endometriosis was investigated by adding the serum of patients with endometriosis into halogenic stem cells. Most of these cells showed morphological changes that resembled endometrial cells and glands, and this differentiation was more intense and faster, the more significant the severity of endometriosis. In addition, differentiated cells expressed ANXA1. These data reveal that there may be inducing factors in women’s blood with endometriosis, highlighting a new theory in endometriosis’s pathogenesis. However, further studies are needed to ratify this and help decipher this substance’s nature and its molecular composition [37].

#### 3.2.4. FPR2/ALX Receptor

##### Expression of FPR2/ALX in Endometriosis

According to the literature, in experimental endometriosis, the expression of FPR2/ALX is more significant in the endometrium and uterus than in the ovary. In addition, this expression is increased in the proestral phase (which corresponds to the ovarian follicular phase) and decreased in the estrous phase (corresponding to the ovulatory stage). In parallel, progesterone administration as 17-OH-progesterone also reduced the expression of FPR2/ALX. Furthermore, in both cross-sectional analysis of endometriosis in humans and experimental endometriosis induced in mice, the FPR2/ALX expression was more significant in endometrial lesions than topical endometrium. These data suggest that this receptor is more expressed in women with endometriosis and even more critical in endometriotic lesions, in addition to being regulated by estrogen and progesterone [26].

## 4. Conclusions

The pro-resolving mediators of inflammation represent potent endogenous factors, allowing the host tissue to maintain homeostasis and prevent chronic inflammatory diseases. Currently, the treatment of endometriosis includes hormonal and anti-inflammatory therapies. However, these therapies are limited due to the high cost, side effects and recurrence of the disease after discontinuing treatment. Among the mediators, LXA4 is the most studied, acting positively in several aspects related to endometriosis progression and maintenance.

According to the reviewed literature, we believe that there is still a knowledge gap regarding the PRM pathways in patients with endometriosis. It is important to note that these substances’ therapeutic potential is evident since the immune response and abnormal inflammatory responses play an essential role in developing, maintaining and progressing this chronic disease.

## Figures and Tables

**Table 1:** Articles captured in the database and grouped according to the main observed mediators. São José, SC—Brazil, 2021.

Database	PRM	Lipoxin	Resolvin	Maresin	ANXA1	FPR
DOAJ	-	2	-	-	3	-
EMBASE	2	92	25	9	81	25
PUBMED	-	20	2	-	4	3
BIREME/LILACS	-	20	2	-	32	3
Total	4	134	39	18	120	31

PRM: pro-resolving mediators; ANXA1: Annexin A1; FPR: formyl peptide receptors.

**Table 2:** Categorization of articles selected by the main authors, journal, design and study target. São José-SC—Brazil, 2021.

Authors	Journal/Year	Study Design	Study Target
Motohashi et al. [26]	Biomed Pharmacother, 2005	Experimental—treatment with LXA4	FPR2/ALX
		Cross-sectional	
Chen et al. [27]	Eur J Obstet Gynecol Reprod Biol, 2009	In vitro: ESC culture—treatment with LXA4 and IL-β	LXA4
Chen et al. [28]	Fertil Steril, 2010	Experimental—treatment with LXA4	LXA4
Xu et al. [29]	Am J Reprod Immunol, 2012	Experimental—treatment with LXA4	LXA4
Gori et al. [30]	Fertil Steril, 2013	Cross-sectional	LXA4
		In vitro: ESC culture—treatment with LXA4	
Wu et al. [19]	British J Pharmacol, 2014	Experimental—treatment with LXA4	LXA4
		In vitro: Endometrioma culture—treatment with LXA4, Boc-2 and IL-1β	LXA4 and FPR2/ALX
		Cross-sectional	
Chen et al. [31]	Fertil Steril, 2014	In vitro: ESC culture—treatment with LXA4	LXA4
		Cross-sectional	
Kumar et al. [32]	PloS ONE, 2014	Experimental—treatment with LXA4	LXA4 and FPR2/ALX
Sobel et al. [33]	Front Endocrinol, 2016	In vitro: ESC culture—treatment with LXA4 and E2	LXA4
Wu et al. [34]	J Obst Gynaecol Res, 2017	In vitro: ESC culture—treatment with LXA4 and IL-1β	LXA4
Wu et al. [6]	Reprod Sci, 2017	Experimental—treatment with LXA4 and Boc-2	LXA4 and FPR2/ALX
		In vitro: Endometrioma culture—treatment with LXA4, Boc-2 and E2	
Dai et al. [35]	Reprod Sci, 2019	Experimental—treatment with LXA4 and Boc-2	LXA4 and FPR2/ALX
		In vitro: Endometrioma culture—treatment with LXA4, MAPK inhibitor and IL-1β	
Li et al. [36]	Chin Med J, 2008	Cross-sectional	ANXA1
Rasheed et al. [37]	J Stem Cel Regen Med, 2010	In vitro: stem cell culture	ANXA1
Paula Jr et al. [38]	J Mol Hist, 2015	Cross-sectional	ANXA1 and FPR1
Volpato et al. [39]	J Reprod Immunol, 2018	Cross-sectional	ANXA1 and FPR2/ALX
Tomio et al. [40]	PloS ONE, 2013	Experimental	RvE2
Dmitrieva et al. [41]	Fertil Steril, 2014	Experimental	RvD1
